# Fatal COVID-19 in a Child with Persistence of SARS-CoV-2 Despite Extensive Multidisciplinary Treatment: A Case Report

**DOI:** 10.3390/children8070564

**Published:** 2021-06-30

**Authors:** Sofia Apostolidou, Theresa Harbauer, Peter Lasch, Daniel Biermann, Maja Hempel, Marc Lütgehetmann, Susanne Pfefferle, Jochen Herrmann, André Rüffer, Konrad Reinshagen, Rainer Kozlik-Feldmann, Anna Gieras, Inga Kniep, Jun Oh, Dominique Singer, Chinedu Ulrich Ebenebe, Robin Kobbe

**Affiliations:** 1 Division of Neonatology and Pediatric Critical Care Medicine, University Children’s Hospital, University Medical Center Eppendorf, 20246 Hamburg, Germany; s.apostolidou@uke.de (S.A.); t.harbauer@uke.de (T.H.); d.singer@uke.de (D.S.); c.ebenebe@uke.de (C.U.E.); 2 Pediatric Intensive Care Medicine, Department of Pediatrics, Clinic Bremen-Mitte, Bremen Hospital Group, 28205 Bremen, Germany; Peter.Lasch@klinikum-bremen-mitte.de; 3 Departments of Pediatric Cardiology and Pediatric Cardiac Surgery, Clinic for Children’s Heart Medicine, University Heart and Vascular Center Hamburg, 20246 Hamburg, Germany; d.biermann@uke.de (D.B.); a.rueffer@uke.de (A.R.); r.kozlik-feldmann@uke.de (R.K.-F.); 4 Institute of Human Genetics, University Medical Center Hamburg-Eppendorf, 20246 Hamburg, Germany; m.hempel@uke.de; 5 Institute for Medical Microbiology, Virology and Hygiene, University Medical Center Hamburg-Eppendorf, 20246 Hamburg, Germany; mluetgehetmann@uke.de (M.L.); s.pfefferle@uke.de (S.P.); 6 Section of Pediatric Radiology, Department of Diagnostic and Interventional Radiology and Nuclear Medicine, University Children’s Hospital, University Medical Center Hamburg-Eppendorf, 20246 Hamburg, Germany; jherrmann@uke.de; 7 Department of Pediatric Surgery, University Medical Center Hamburg-Eppendorf, 20246 Hamburg, Germany; k.reinshagen@uke.de; 8 Department of Immunology, University Medical Center Hamburg-Eppendorf, 20246 Hamburg, Germany; a.gieras@uke.de; 9 Institute of Legal Medicine, University Medical Center Hamburg-Eppendorf, 22529 Hamburg, Germany; inga.kniep@uke.de; 10 Department of Pediatric Nephrology, University Children’s Hospital, University Medical Center Hamburg-Eppendorf, 20246 Hamburg, Germany; j.oh@uke.de; 11 First Department of Medicine, Division of Infectious Diseases, University Medical Center Hamburg-Eppendorf, 20246 Hamburg, Germany

**Keywords:** coronavirus disease 2019 (COVID-19), children, DExH-BOX helicase 30 (DHX30), extracorporeal life support (ECLS), Impella

## Abstract

Critical Coronavirus disease 2019 (COVID-19) developed in a 7-year-old girl with a history of dystrophy, microcephaly, and central hypothyroidism. Starting with gastrointestinal symptoms, the patient developed severe myocarditis followed by progressive multiple organ failure complicated by *Pseudomonas aeruginosa* bloodstream infection. Intensive care treatment consisting of invasive ventilation, drainage of pleural effusion, and high catecholamine therapy could not prevent the progression of heart failure, leading to the implantation of venoarterial extracorporeal life support (VA-ECLS) and additional left ventricle support catheter (Impella^®^ pump). Continuous venovenous hemofiltration (CVVH) and extracorporeal hemadsorption therapy (CytoSorb^®^) were initiated. Whole exome sequencing revealed a mutation of unknown significance in DExH-BOX helicase 30 (*DHX30*), a gene encoding a RNA helicase. COVID-19 specific antiviral and immunomodulatory treatment did not lead to viral clearance or control of hyperinflammation resulting in the patient’s death on extracorporeal life support-(ECLS)-day 20. This fatal case illustrates the potential severity of pediatric COVID-19 and suggests further evaluation of antiviral treatment strategies and vaccination programs for children.

## 1. Introduction

Symptomatic COVID-19 in children appears mostly mild or moderate, although Multisystem Inflammatory Syndrome in children (MIS-C) is the most frequent presentation among critically ill children with SARS-CoV-2 infection [1,2].

Since the beginning of the pandemic, only a few deaths in children with COVID-19 have been reported [3]. Analysis of child COVID-19 mortality in seven countries (USA, UK, Italy, Germany, Spain, France, and South Korea) up to February, 2021, show a low rate at 0.17 per 100,000 population. This comprises 0.48% of the estimated total mortality from all causes in a normal year, relatively more frequent in older children than younger age groups. No clear evidence of a trend of increasing mortality was detectable, but additional deaths have clearly occurred in children and young people during periods of high community transmission [4,5]. Currently, we are just beginning to understand whether specific risk factors or pre-existing medical conditions, as described in adults, are associated with severe disease and MIS-C in children [6,7].

## 2. Case Report

A seven-year-old female patient with headache, loss of appetite, abdominal pain, and vomiting for three days in the absence of cough, fever, or diarrhea was admitted to the regional hospital. The patient’s medical history was notable for late preterm birth, central hypothyroidism, failure to thrive (body weight 18 kg, <1. percentile), and recurrent respiratory tract infections. No laboratory tests to exclude primary immunodeficiency had been conducted previously. She attended a regular primary school. On admission, the patient was alert and showed mild signs of dehydration, but normal findings on abdominal, respiratory, cardiovascular, and neurologic examination. Laboratory tests (see Supplementary Material) revealed moderate signs of infection with elevated white blood cells, neutrophils, and C-reactive protein (CRP). The following day, she presented with increasing abdominal pain and distension, decreased peristaltic, free intraperitoneal fluid, and bilateral pleural and pericardial effusions on ultrasound examination. SARS-CoV-2 PCR from the nasopharyngeal swab sample was negative at this point.

### 2.1. Respiratory and Cardiovascular System

After referral to the municipal hospital on day 6 of illness, echocardiography revealed signs of myocarditis with significant impairment of the myocardial function (fractional shortening, FS 18%) and increased pericardial effusion. Due to increasing respiratory distress, non-invasive ventilation was initiated, and drainage of right pleural effusion mobilized 500 mL clear fluid. However, rapidly advancing respiratory failure led to intubation, and now SARS-CoV-2 PCR from tracheal aspirate was positive on day 7. The following days were marked by progressive multiple organ failure with acute respiratory distress syndrome (ARDS), cardiac failure requiring high-dose catecholamine therapy, and renal failure with oliguria and impaired glomerular filtration rate (GFR). Hypoxemic respiratory failure despite intensive, invasive ventilation therapy developed and persistent hypotension, due to cardiac failure, indicated a rescue therapy with ECLS. The patient was transferred to our University Medical Center, and with progressive cardiac failure (FS 10%) despite the maintenance of catecholamine therapy (milrinone, epinephrine, and norepinephrine), an ECLS, Impella^®^ Heart Pump (Abiomed, Aachen Germany) was inserted via the ascending aorta for unloading of the left ventricle on day 20 after the onset of symptoms [8]. Impella^®^ was discontinued, due to cardiac recovery after eight days of support and administration of Levosimendan for 48 hours. A myocardial biopsy confirmed the diagnosis of acute lymphocytic myocarditis—importantly, SARS-CoV-2 PCR from myocardial tissue was negative. Computed tomography of the chest on day 19 demonstrated diffuse, bilateral ground glass opacities with pulmonary consolidations, visible intralobular lines, and dilated subsegmental vessels. Application of pleural drainage continuously mobilized significant amounts of effusion over the next days. Notably, despite pronounced radiologic signs of pneumonia, a bronchoscopy performed on day 36 showed no significant pathology of the bronchopulmonary system, while lung biopsy showed diffuse alveolar damage, compatible with ARDS.

### 2.2. Anticoagulation and Transfusion Therapy

Intravenous therapeutic heparin infusion was conducted during ECLS therapy. Several blood products and anticoagulant drugs (fresh frozen plasma (FFP), platelet concentrates (PC), red blood cell concentrates (RBCC), PPSB, recombinant factor VIIa, and tranexamic acid) were administered, due to bleedings from cannula within the first 24 h after initiation, and several transfusions of FFP, PC, and RBCC were performed during ECLS therapy.

### 2.3. Renal Therapy

The patient developed acute kidney injury with oligo-anuria, possibly related to SARS-CoV-2 renal tropism, which represents a marker for multiorgan failure with a need for extracorporal therapies in adults [9,10]. Diuretic therapy (furosemide and ethacrynic acid) was started on day 28 and supported by continuous venovenous hemofiltration (CVVH) from day 31. Kidneys appeared not enlarged, but signal intense on ultrasound, a biopsy was not performed.

### 2.4. Infection and Inflammation

**Figure 1:**
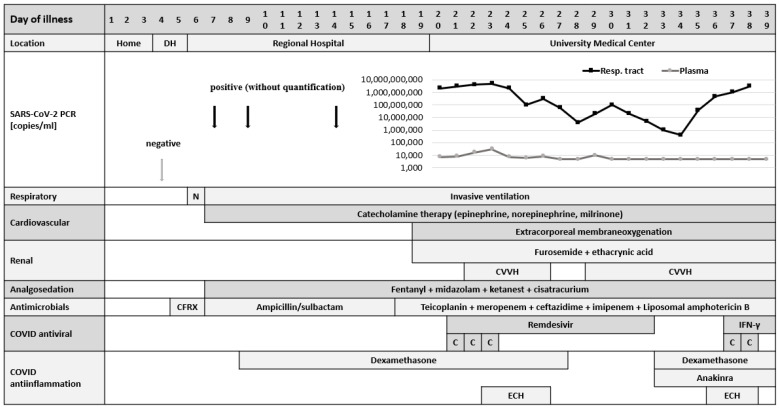
Clinical course with SARS-CoV-2 Viral load in nasophyryngeal swaps and blood. Abbreviations: DH = district hospital, N = non-invasive ventilation, CVVH = continuous veno-venous hemofiltration, CFRX = cefuroxime, C = convalescent plasma, ECH = extracorporeal hemadsorption, IFN-γ = interferon-γ.

Initial antimicrobial treatment with a cephalosporin was switched to ampicillin plus sulbactam, due to persisting laboratory signs of a bacterial infection with severe lymphopenia and a high neutrophil-lymphocyte ratio. In the absence of fever, a mandatory clinical sign of the diagnosis of MIS-C, a *Pseudomonas aeruginosa* bloodstream infection, was detected on day 21, and treated according to resistance testing. Due to the disease severity and persisting high SARS-CoV-2 RNA load in tracheal aspirate and blood measured by cobas 6800/8800 RT-PCR assay [11], the nucleotide analog prodrug remdesivir (5 mg/kg, followed by 2.5 mg/kg for ten days), an inhibitor of the viral RNA-dependent, RNA polymerase, was administered. Notably, the presence of a variant of concern was excluded [12]. Importantly, at different time points, neither IgM nor IgG antibodies against SARS-CoV-2 glycoprotein (S) or nucleoprotein (N) could be detected in two different serological assays (Elecsys, Roche, USA, DiaSorin Liaison, Germany). To boost antiviral activity, a total of 5 units (>1000 mL) of convalescent plasma (mean SARS-CoV-2 IgG 67.6 AU/mL, range 36.5–96.6) were transfused, but could not induce significant viral RNA clearance (Figure 1).

**Figure 2:**
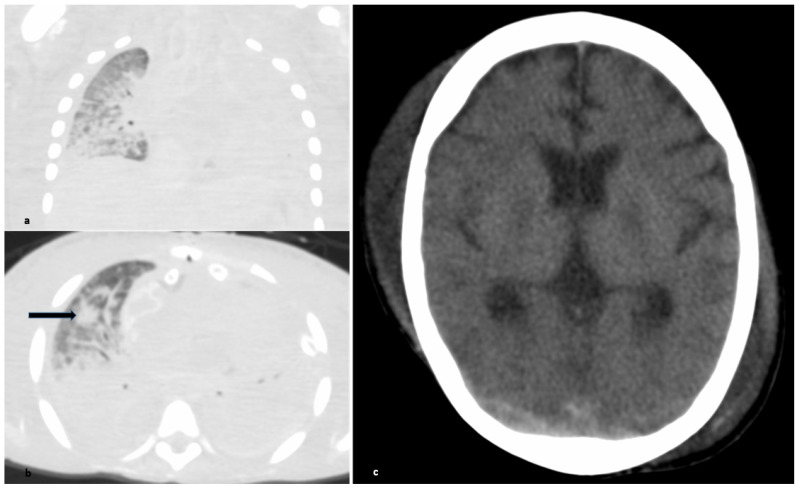
Postmortem computed tomography (PMCT): (**a**) Coronary CT image (lung section); (**b**) axial CT image (lung section); patchy area of consolidation ventral (blue arrow); (**c**) axial CT image (section of head).

Despite the absence of fever, severe illness with multisystemic inflammation and especially COVID-19 associated myocarditis triggered MIS-C-like-disease treatment early in the disease course with repeated high-dose intravenous immunoglobulins and dexamethasone administration, followed by escalating doses of the interleukin-1 receptor antagonist anakinra to control inflammation. To further control systemic inflammatory response syndrome (SIRS), extracorporeal hemadsorption therapy (CytoSorb^®^, Cytosorbents, Berlin, Germany) was initiated on day 32, which temporally improved the clinical picture and reduced serum levels of IL-6, CRP, and Ferritin. Nevertheless, the patient‘s circulatory system was continuously dependent on ECLS and inotropic support. As further deterioration after 14 days on ECLS with the persistence of hyperinflammation and ongoing SARS-CoV-2 replication seemed inevitable, we initiated antiviral interferon-γ treatment considering a monogenetic disease with a candidate gene mutation in DHX30. Despite pronounced intensive care treatment, the patient died on day 20 on ECLS. Unfortunately, an autopsy was refused by the parents; therefore, additional exploration was only available by postmortem CT (PMCT), shown to be particularly useful in the current pandemic situation [13,14]. In our case, PMCT showed complete shadowing of the left lung with extensive consolidations and positive broncho-pneumogram, diffuse ground glass opacities, and extensive consolidations (Figure 2). The brain showed cerebral edema, while there were no signs of intracerebral bleeding.

### 2.5. Genetic Examination

Before the COVID-19 infection, multiple genetic tests were performed to determine a genetic cause of primary microcephaly and dystrophy, revealing a normal female karyotype, inconspicuous results in Array-CGH and panel analysis. Rapid Trio-Whole-Exome-Sequencing (WES) showed a heterozygote *DHX30* variant (c.675T>A, p. (Ser225Arg)), also present in the healthy mother. To further explore the role of DHX30 in COVID-19, in vitro functional testing is underway.

## 3. Discussion

We report a case of critical COVID-19 in a 7-year-old girl with dystrophy, central hypothyroidism. Despite maximal intensive care treatment, including organ replacement procedures (ECLS, Impella^®^, hemofiltration, and hemabsorption therapy) and antiviral, as well as immunomodulatory therapies, the clinical course without viral clearance progressively worsened, leading to death 39 days after onset of disease and 20 days after initiation of ECLS.

Gastrointestinal symptoms marked the beginning of the illness, not fever or respiratory symptoms; this has been repeatedly described as a potential risk factor for critical COVID-19, especially in children with MIS-C [15]. Severe myocarditis leading to heart failure in our patient responded to the initiated therapy with ECLS and left-ventricular Impella^®^ Heart Pump. The leading cause of mortality in adults is respiratory failure. Despite the recovery of cardiac function in our patient, the developing ARDS, supportively treated with ECLS and gentle ventilation, could not restore lung function. We assume persistent hyperinflammatory syndrome as the leading cause of pulmonary and multiorgan failure concluding that mortality might be related to virologically driven hyperinflammation. Intravenous off-label use of remdesivir was started with 5 mg/kg on the first day, followed by 2.5 mg/kg daily, as suggested from Ebola treatment trials, and suggested for compassionate use in children and therapeutic drug monitoring of the prodrug. Elimination of remdesivir by hemadsorption using CytoSorb^®^ devices has been reported [16]. Still, we structured procedures and drug application in a way so that the metabolite GS-441524 could be measured during ECLS and hemofiltration by the UHPLC-MS/MS method revealing target levels [17].

Twenty days after onset of symptoms, there were still no anti-SARS-CoV-2 antibodies detectable. Therefore, we used passive antibody transfer and transfused convalescent plasma on subsequent days without achieving viral clearance. Unfortunately, specific monoclonal antibodies directed against SARS-CoV-2 spike protein were not available in Germany.

Corticosteroids as immunosuppressive therapy in hyperinflammation syndrome have been shown to reduce mortality in adults, and selected IL-1 blockade with high dose anakinra has been recommended as a therapeutic option. In our hands, even in combination with Cytosorb^®^, this approach was not sufficient to establish control inflammation and thereby circulation. While the presence of anti-interferon autoantibodies was excluded, this unusual clinical development with the hallmark of high viral RNA loads and viremia gave rise to consider a pathogenic monogenetic disease [18,19]. Rapid Trio-WES could not detect an inborn error in the type I interferon-pathway [20], but detected a candidate heterozygote *DHX30* variant (c.675T>A, p. (Ser225Arg)). *DHX30* encodes for an RNA helicase that uses ATP hydrolysis to unwind RNA secondary structures and is required for most aspects of RNA metabolism and function [21]. Importantly, DHX30 interacts with ZAP (zinc-finger antiviral protein) and has been shown to restrict SARS-CoV-2. Therefore, a mutation in this highly conserved region might negatively affect RNA virus defense by insufficient interferon-γ [22]. Noteworthy, in the presence of severe lymphopenia, no natural killer cells or plasmacytoid dendritic cells could be detected. Acknowledging the possibility of interferon-γ deficiency [23], while judging the current situation of the patient doomed to deterioration, we decided to treat the patient with intravenous interferon-γ, hoping to boost antiviral potency and considering a worsening of the hyperinflammation. After administration of interferon-γ in combination with convalescent plasma and high dose anakinra, viral RNA loads and hyperinflammation increased. Unfortunately, the patient died three days later with fulminant multiorgan-failure.

## 4. Conclusions

Although rare, COVID-19 with hyperinflammation in children can result in fatal organ damage despite maximal intensive care. Clinical trials testing antiviral strategies in children, and even more important, SARS-CoV-2 vaccination programs are urgently needed.

## Supplementary Materials

The following are available online at https://www.mdpi.com/article/10.3390/children8070564/s1. Supplementary: laboratory data.
